# Development of an efficient *Agrobacterium*-mediated transformation method and its application in tryptophan pathway modification in *Catharanthus roseus*

**DOI:** 10.5511/plantbiotechnology.23.0819a

**Published:** 2023-12-25

**Authors:** Hiroaki Kisaka, Dong Poh Chin, Tetsuya Miwa, Hiroto Hirano, Sato Uchiyama, Masahiro Mii, Mayu Iyo

**Affiliations:** 1 Biosolutions Development Section, Biosolutions Labs, Research Institute for Bioscience Products & Fine Chemicals, Ajinomoto Co., Inc., 1-1 Suzuki-cho, Kawasaki-ku, Kawasaki-shi, Kanagawa 210-8681, Japan; 2 Center for Environment, Health and Field Sciences, Chiba University, 6-2-1 Kashiwanoha, Kashiwa, Chiba 277-0882, Japan

**Keywords:** *Agrobacterium rhizogenes A13*, *Catharanthus roseus*, transformation, tryptophan, vinca alkaloid

## Abstract

The biosynthetic pathway of *Catharanthus roseus* vinca alkaloids has a long research history, including not only identification of metabolic intermediates but also the mechanisms of inter-cellular transport and accumulation of biosynthesized components. Vinca alkaloids pathway begins with strictosidine, which is biosynthesized by condensing tryptamine from the tryptophan pathway and secologanin from the isoprenoid pathway. Therefore, increasing the supply of precursor tryptophan may enhance vinca alkaloid content or their metabolic intermediates. Many reports on the genetic modification of *C. roseus* use cultured cells or hairy roots, but few reports cover the production of transgenic plants. In this study, we first investigated a method for stably producing transgenic plants of *C. roseus*, then, using this technique, we modified the tryptophan metabolism system to produce transgenic plants with increased tryptophan content. Transformed plants were obtained by infecting cotyledons two weeks after sowing with *Agrobacterium* strain A13 containing a plant expression vector, then selecting with 1/2 B5 medium supplemented with 50 mg l^−1^ kanamycin and 20 mg l^−1^ meropenem. Sixty-eight regenerated plants were obtained from 4,200 cotyledons infected with *Agrobacterium*, after which genomic PCR analysis using *NPTII*-specific primers confirmed gene presence in 24 plants with a transformation rate of 0.6%. Furthermore, we performed transformation into *C. roseus* using an expression vector to join *trpE8* and *aroG4* genes, which are feedback-resistant mutant genes derived from *Escherichia coli*. The resulting transformed plants showed exactly the same morphology as the wild-type, albeit with a marked increase in tryptophan and alkaloids content, especially catharanthine in leaves.

## Introduction

*Catharanthus roseus* biosynthesizes many terpenoid-indole alkaloids (TIAs) and over 130 compounds to date have been isolated and identified (Heijden et al. 2004). Many TIAs are pharmaceutically important compounds and widely used. Ajmalicine, which is biosynthesized by *C. roseus*, is used as an anti-hypertensive agent, and vinblastine and vincristine are anticancer agents to treat lymphoma and leukemia (Jordan et al. 1991). Recent research shows vinblastine and vincristine can be semi-synthesized using catharanthine and vindoline as precursors (Ishikawa et al. 2009). However, due to the complex structure of TIAs, complete synthesis using chemical synthesis methods can be difficult (Yang and Stöckigt 2010). Catharanthine, vindoline, and ajmalicine are the main alkaloids in *C. roseus*, yet given the important social significance of increasing the content of these alkaloids, no experiments showing a dramatic increase have been reported.

The biosynthetic pathway (TIA pathway) for these key compounds begins with strictosidine, formed by the condensation of tryptamine and secologanin (Guirimand et al. 2010). Tryptamine is derived from shikimate and tryptophan biosynthetic pathways (Zhao et al. 2013), while secologanin is derived from the 2-C-methyl-delythritol 4-phosphate and terpenoid biosynthetic pathways (Miettinen et al. 2014). Downstream biosynthetic pathways, such as vindoline, catharanthine, and ajmalicine, are unique to *C. roseus* and have not been discovered in other organisms (Murata et al. 2008). These components are biosynthesized at very low levels (Glenn et al. 2013), and there is an urgent need for further research to increase TIA production in *C. roseus* using genetic engineering techniques.

Anthranilate synthase (AS) is an enzyme that catalyzes chorismate to anthranilate and likely plays an important role in regulating the indole pathway (Hughes et al. 2004). In *E. coli*, studies show the trpE and trpD complex can catalyze chorismate into anthranilic acid (Oppenheim et al. 1980). The aroG constitutes a 3-deoxy-D-arabino-heptulosonate-7-phosphate synthase that is implicated in tyrosine, phenylalanine, and tryptophan metabolism. The *trpE8* and *aroG4* genes used in this study are feedback-resistance genes of *trpE* and *aroG*, respectively. Alkaloids biosynthesized in *C. roseus* are thought to be transported and stored in heteromorphic cells and epidermal cells (Facchini and De Luca 2008), and transporters involved in alkaloid transport have also been reported. CrTPT2 is a type of ABC transporter that transports catharanthine to the epidermal surface to secrete it (Yu and De Luca 2013). In addition, three nitrate/peptide family (NPF) transporters, CrNPF2.4, CrNPF2.5, and CrNPF2.6, contribute to intracellular transport of multiple iridoid intermediates (Larsen et al. 2017). Further, strictosidine, formed following secologanin and tryptamine binding in the cytosol, is exported to the cytosol via the tonoplast-localized NPF transporter CrNPF2.9 (Payne et al. 2017). Thus, there is little information on alkaloid transporters in *C. roseus*. We used the Nt-JAT2 transporter (Morita et al. 2009), which transports nicotine, an alkaloid found in tobacco. Nt-JAT2 transporter accumulates alkaloids in vacuoles and is presumed to have a different function from CrTPT2 and the CrNPF family. Since many vinca alkaloids are thought to be stored in vacuoles, we investigated the usefulness of the Nt-JAT2 transporter.

In this study, we established a transformation system using *Agrobacterium* to convert *C. roseus* to analyze its effect on alkaloid biosynthesis by increasing the amount of tryptophan, a precursor of alkaloid biosynthesis. Finally, we obtained transformed plants with elevated tryptophan levels. These transgenic plants were confirmed to have increased levels of alkaloids, such as catharanthine.

## Materials and methods

### Construction of the expression vector

We used the plant expression vector pRI201-AN (Takara Bio Inc.). *E. coli*-derived genes encoding the α subunit of AS, which was the feedback-resistance gene (*trpE8*) (Ramos et al. 2008), the β subunit of AS (*trpD*) and 3-deoxy-D-arabino-heptulosonate-7-phosphate synthase which was also the feedback-resistant mutant gene (*aroG4*) (Doroshenko et al. 2010; Kikuchi et al. 1997) were inserted into the expression vector respectively. In addition, *Nt-JAT2* gene isolated from tobacco was used to investigate the usefulness of vinca alkaloid transporters.

**Figure 1:**
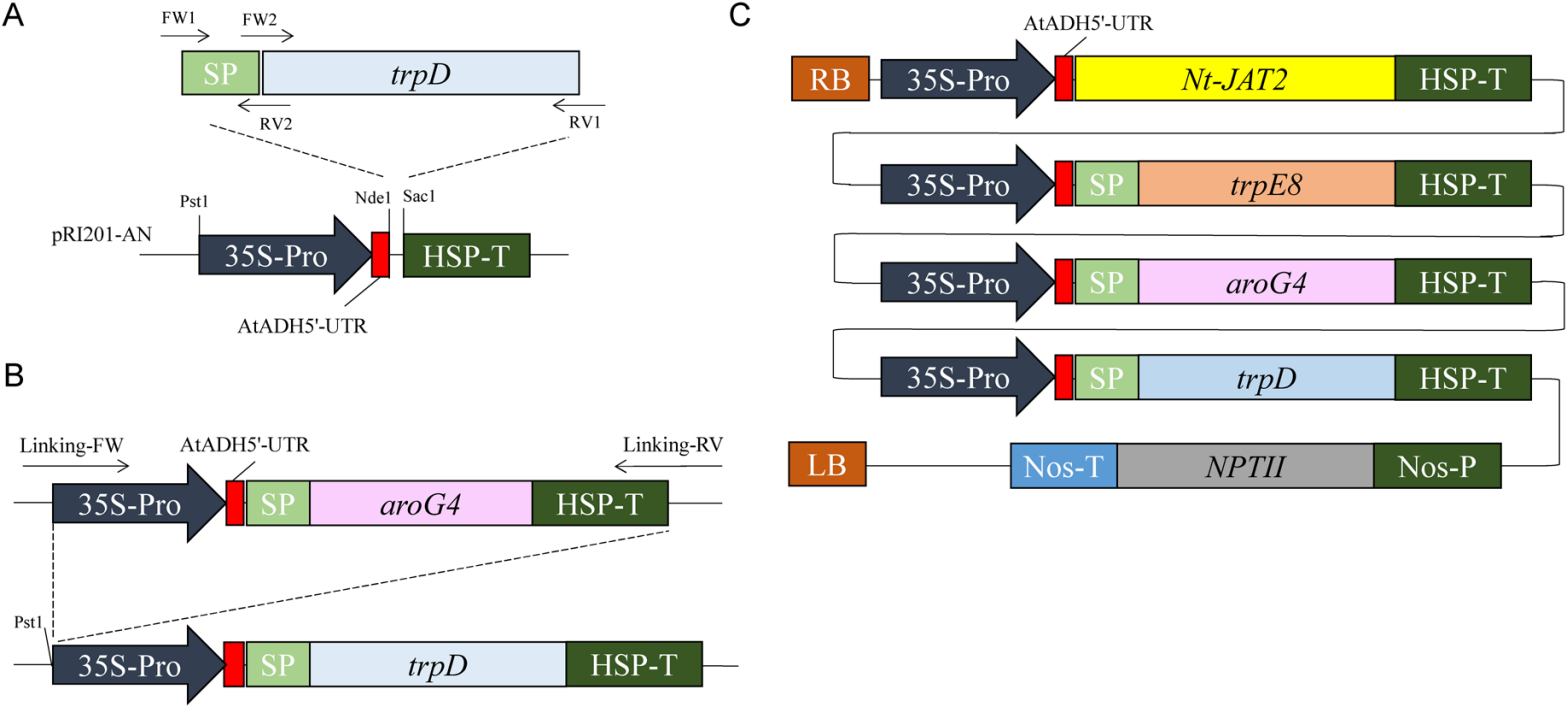
Figure 1. A JAED genes cluster vector created in this experiment. (A) A method for linking the aldolase signal sequence and *trpD* gene by PCR. Similarly, signal sequences were added to *trpE8* gene and *aroG4* gene. (B) Ligation of four expression cassettes. The region from the promoter to the terminator was used as one expression cassette. Following amplification by PCR using the primers Linking-FW and Linking-RV (Table 1), these four expression cassettes were sequentially joined at the PstI site of pRI201-AN. (C) The JAED expression vector connecting four expression cassettes was used in this experiment. It contains mutant genes, the *trpE8* gene, *aroG4* gene, and *trpD* gene derived from *E. coli* that is feedback-resistant in the tryptophan metabolic system and an alkaloid transporter *Nt-JAT2* gene isolated from tobacco. SP: signal peptide sequence of Aldolase (GenBank accession no. GU723954, 174 bp from N-terminal), AtADH5′-UTR: 5′non-coding region of *Arabidopsis* alcohol dehydrogenase (translational enhancer), 35S-Pro: cauliflower mosaic virus promoter for expression of target gene in plant, *NPTII*: neomycin phosphotransferase (Selectable marker gene in plant), HSP-T: HSP terminator for expression of foreign genes in plants, Nos-P: NOS promoter, Nos-T: NOS terminator, RB: right border sequence, LB: left border sequence.

The *trpE8* gene was obtained by a PCR reaction using genomic DNA of *E. coli* strain TrpE8 with the *trpE8* mutant gene as a template and primers 5′-ATGCAAACACAAAAACCGACTCTC-3′ and 5′-TCAGAAAGTCTCCTGTGCATGATG-3′. For the PCR reaction, we used Prime Star max DNA Polymerase (Takara Bio Inc.) and performed amplification in 35 cycles of denaturation at 98°C for 10 s, annealing at 55°C for 5 s, and extension at 72°C for 50 s. Similarly, primers 5-ATGGCTGACATTCTGCTGCTCGATAATATCGACTC-3 and 5′-TTACCCTCGTGCCGCCAGTGCGGTGACTCTGTC-3′ were used to amplify the *trpD* gene. In addition, the *aroG4* gene was also obtained by a PCR reaction using genomic DNA of *E. coli* strain AB3257. The primers 5′-ATGAATTATCAGAACGACGATTTA-3′ and 5′-TTACCCGCGACGCGCTTTTACTGC-3′ were used to amplify the *aroG4* gene. Furthermore, because of the synthesis of tryptophan is performed in plant chloroplasts, we used the aldolase signal sequence (GenBank accession no. GU723954, 174 bp from the N-terminus, Table 1), which is a translocation sequence to the chloroplast (Mininno et al. 2012). This signal sequence was joined to the N-terminus of each of the three aforementioned genes (*trpE8*, *aroG4*, and *trpD*) using the primers presented in Table 1. The aldolase signal sequence was amplified using PCR and the primers Ald-Signal Peptide FW1 and Ald-trpD-RV2, and *trpD* was amplified using PCR and the primers Ald-trpD-FW2 and Ald-trpD-RV1. After purification, the PCR products were combined and used as a template, and PCR was performed using the primers FW1 and RV1 to join the two genes, as presented in Figure 1A. An aldolase signal sequence was similarly added to *trpE8* and *aroG4*. Each gene was inserted into the binary vector pRI201-AN (Takara Bio Inc.), which was digested using the restriction enzymes NdeI and SacI, and each gene was joined to this site using an In Fusion HD Cloning Kit (Takara Bio Inc.). *Nt-JAT2* was synthesized in reference to GenBank accession no. AB922128 and joined to the binary vector pRI201-AN. The region from the promoter to the terminator was used as one expression cassette. Following amplification by PCR using the primers Linking-FW and Linking-RV (Table 1), these four expression cassettes were sequentially joined using the PstI site of pRI201-AN, as presented in Figure 1B. Finally, an expression vector was constructed and termed JAED (Figure 1C). The vector was previously transformed into *Agrobacterium rhizogenes* strain A13 (Nakano et al. 1994). Using the Green Fluorescent Protein gene as a vector control, it was similarly joined to pRI201-AN, then transformed into *Agrobacterium rhizogenes* strain A13. These were then used to prepare transformed plants using *C. roseus*.

**Table 1:** Table 1. List of primers used to construct the expression vectors and aldolase signal sequence information.

Primer name	Sequence
Ald-Signal Peptide FW1	5′-TCTTCACTGTTGATACATATGGCTATGTCTAATACTTCT-3′
Ald-trpD-FW2	5′-CCTCCTAAGACTCCTATTTCTATGGCTGACATTCTGCTGCTC-3′
Ald-trpD-RV2	5′-GAGCAGCAGAATGTCAGCCATAGAAATAGGAGTCTTAGGAGG-3′
Ald-trpD-RV1	5′-TCATCTTCATAAGAGCTCTTACCCTCGTGCCGCCAGTGC-3′
Ald-aroG-FW2	5′-CCTAAGACTCCTATTTCTATGAATTATCAGAACGAC-3′
Ald-aroG-RV2	5′-GTCGTTCTGATAATTCATAGAAATAGGAGTCTTAGG-3′
Ald-aroG-RV1	5′-TCATCTTCATAAGAGCTCTTACCCGCGACGCGCTTTTAC-3′
Ald-trpE-FW2	5′-CCTAAGACTCCTATTTCTATGCAAACACAAAAACC-3′
Ald-trpE-RV2	5′-GGTTTTTGTGTTTGCATAGAAATAGGAGTCTTAGG-3′
Ald-trpE-RV1	5′-TCATCTTCATAAGAGCTCTCAGAAAGTCTCCTGTGCATG-3′
Nt-JAT2-F	5′-TCTTCACTGTTGATACATATGGAATCACCATTGCTCGAT-3′
Nt-JAT2-R	5′-TCATCTTCATAAGAGCTCTCACGCGTAAATCTTAGCTCT-3′
Linking-FW	5′-ACGACGGCCAGTGCCAAGCTTCTGCAGGTCCCCAGATTAGCCTTTTCA-3′
Linking-RV	5′-TGAAAAGGCTAATCTGGGGACCTTATCTTTAATCATATTCC-3′
Aldolase signal peptide information	
Amino acid-amino acid sequence (nucleotide sequence)	MAMSNTSALASKLLPSCKPHQPTLTFFSPSTTCQKKPRSSRPISAAVHVTQPPKTPIS (ATGGCTATGTCTAATACTTCTGCTCTTGCTTCTAAGCTTCTTCCTTCTTGTAAGCCTCATCAACCTACTCTTACTTTTTTTTCTCCTTCTACTACTTGTCAAAAGAAGCCTAGATCTTCTAGACCTATTTCTGCTGCTGTTCATGTTACTCAACCTCCTAAGACTCCTATTTCT)

The ligation of the aldolase signal sequence and target gene is presented in Figure 1A. FW1, FW2, RV1, and RV2 correspond to their positions presented in Figure 1A.

### Sowing of *C. roseus* seeds and cotyledons preculture

Seeds of *C. roseus* (cultivar, Pacifica XP Really Red, Takii Co., Ltd.) were soaked in 70% (v/v) ethanol for 1 min, after which ethanol was removed and seeds were then soaked in 2% (v/v) sodium hypochlorite for 15 min. Sterilized seeds were then washed three times with sterilized water and seeded on 1/2 B5 (Gamborg et al. 1968) solid medium supplemented with 3% (v/v) sucrose and 0.5% (v/v) gellan gum (Nacalai tesque Inc.) by aseptic manipulation. The seeds were cultured for 2 weeks at 25°C and 16 h photoperiod condition. Germinated cotyledons were excised while taking care to exclude meristems, placed on the same 1/2 B5 agar medium, and similarly precultured at 25°C for 24 h under 16 h photoperiod conditions.

### *Agrobacterium* infection, sterilization, and selection

*Agrobacterium rhizogenes* strain A13 were inoculated into 2 ml of YEP liquid medium (bacto peptone 10 g l^−1^, yeast extract 10 g l^−1^, NaCl 5 g l^−1^, pH 7.0) and cultured at 28°C for 24 h. Grown *Agrobacterium* was diluted approximately 100-fold with sterile water (OD600=0.01–0.05), after which 30 mg l^−1^ acetosyringone (3′5′-dimethoxy-4′-hydroxyacetophenone, Sigma-Aldrich) was added and mixed well. Precultured cotyledons were transferred to prepared *Agrobacterium* suspension and soaked for 10 min, after which cotyledons were removed with sterilized filter paper, transferred to 1/2 B5 agar medium, and cultured at 25°C for 16 h under photoperiod conditions for 24 h. After 24 h, cotyledons were transferred to 1/2 B5 medium containing 20 mg l^−1^ meropenem (FUJIFILM Wako Pure Chemical Industries, Ltd. Corporation) and *Agrobacterium* was removed for five days. Cotyledons were then transferred to 1/2 B5 medium containing 50 mg l^−1^ kanamycin (Nacalai Tesque Inc.) and 20 mg l^−1^ meropenem to initiate selection. After one week post infection with *Agrobacterium*, we observed callus formation near the cotyledon incision, and then one month later, confirmed root differentiation from the callus of some cotyledons. When the cotyledon culture in which rooting was observed was continued, shoots were regenerated from the callus in a different part from the regenerated roots. The resulting shoots were cut from the stem base and transplanted onto 1/2 B5 agar medium containing 100 mg l^−1^ of kanamycin to induce rooting. Shoots never regenerate directly from the root. Completely regenerated plants were acclimatized to the soil and continued to be cultivated.

### Analysis of transgenes in regenerated plants

DNA was extracted from regenerated plant leaves using the DNeasy Plant Mini Kit (Qiagen). *NPTII* gene-specific primers (5′-AGGATCTCCTGTCATCTCACCTT-3′ and 5′-CTCTTCAGCAATATCACGGGTAG-3′) were utilized to confirm the presence of the *NPTII* gene in the genomic DNA. PCR was performed using KOD one PCR Master Mix (TOYOBO Co., Ltd.) under 35 cycles of denaturation at 98°C for 10 s, annealing at 55°C for 5 s, and extension at 68°C for 10 s. For total RNA extraction, similarly, regenerated plant leaves were used with the RNeasy Plant Mini Kit (Qiagen). In addition, in this extraction step, DNase I treatment was performed to prevent DNA contamination. Furthermore, cDNA was prepared with the Prime Script RT reagent kit (Takara Bio Inc.) using 1 µg of extracted total RNA. To verify the quality of the cDNA, expression levels of the ubiquitin gene were evaluated. The ubiquitin gene-specific primers 5′-GCGGATTACAACATTCAAAAGGAG-3′ and 5′-GTGAGAGTCTTGACAAAGATTTGC-3′ were used and PCR was performed using KOD one PCR Master Mix with 35 cycles of denaturation at 98°C for 10 s, annealing at 55°C for 5 s, and extension at 68°C for 10 s. To confirm expression of introduced genes, we performed RT-PCR analysis and each gene-specific primer set was used for analysis. Specifically, 5′-CCCGAAAAGCGATCCATAAG-3′ and 5′-CAGCTCATCAGGTCAGCGAG-3′ for *aroG4* gene analysis, 5′-CGTAGTGCGTTTGTTGCAAA-3′ and 5′-GCAATCGGGTAGATCTCAATC-3′ for *trpE8* gene, 5′-ATCAGGCGATTGTCGAGGCTTAC-3′ and 5′-AGGCCAGCGTTTGTTCCA-3′ for the *trpD* gene, and primer sets of 5′-GGGCAAGTACACATGCTTGG-3′ and 5′-GGATATAGCTGCCCCGGTTA-3′ were used for the *Nt-JAT2* gene. The PCR conditions and polymerase used were as above mentioned.

### Histochemistry and microscopy

GFP visualization and documentation were performed using a Leica S APO Stereo Zoom Microscope (Leica microsystems). GFP detection was using a fluorescence adapter for a stereo microscope and a Royal Blue filter (excitation light wavelength 440–460 nm, fluorescence wavelength 500 nm).

### Amino acid analysis

We cut and sampled 100 mg leaf disks from the 4th, 5th, and 6th leaves from the growing point of regenerated plants (T_0_ generation). The sampled leaves were immediately frozen in liquid nitrogen and ground using a mixer mill MM300 (Verder Scientific) before adding 500 µl of 80% (v/v) ethanol. After shaking at 2,000 rpm for 30 min at room temperature, we ran centrifugation at 12,000 rpm for 20 min and the supernatant was transferred to a new tube. We then added 500 µl of 80% (v/v) ethanol to the remaining precipitate and performed ethanol extraction three times in total. Extracts were then dried in a vacuum evaporator. After adding 400 µl of sterilized water and dissolving, we added 200 µl of chloroform and mixed well to remove proteins. We then centrifuged the solution at 12,000 rpm for 10 min at room temperature and transferred the supernatant to another new tube. We added 20 µl of 0.2-N hydrochloric acid to 180 µl of purified solution, mixed well, and filtered the solution using an Ultra-free-MC 0.22-µm filter (Merck Millipore). The filtrate was analyzed for amino acid content using a HITACHI high-speed amino acid analyzer, L8800 (Hitachi High-Tech Corporation).

### Alkaloid analysis

We cut and sampled 100 mg leaf disks from the 4th, 5th, and 6th leaves from the growing point of regenerated plants (T_0_ generation). The sampled leaves were immediately frozen in liquid nitrogen, ground using a Mixer Mill MM300, and added to 500 ml of 99% (v/v) methanol. After shaking at 30°C at 2,000 rpm for 2 h, the mixture was centrifuged at 12,000 rpm at room temperature for 20 min, and the supernatant was transferred to another tube. For extraction, the supernatant was filtered using an Ultra-free-MC 0.22-µm filter, and the filtrate was used as a sample. For UPLC (ACQUITY UPLC H-Class PLUS, Waters) analysis, an ACQUITY UPLC BEH C18 1.7 µm 2.1×100 mm column (Waters) was used with 30% (v/v) methanol and 0.1 M phosphoric acid (pH 2.0) at 1 ml min^−1^. The column temperature was 40°C and detection was performed by UV (281 nm) absorbance. Catharanthine, vindoline, and ajmalicine, which served as standards in this experiment, were purchased from Sigma-Aldrich.

## Results

### Study of *Catharanthus roseus* transformation system

**Figure 2:**
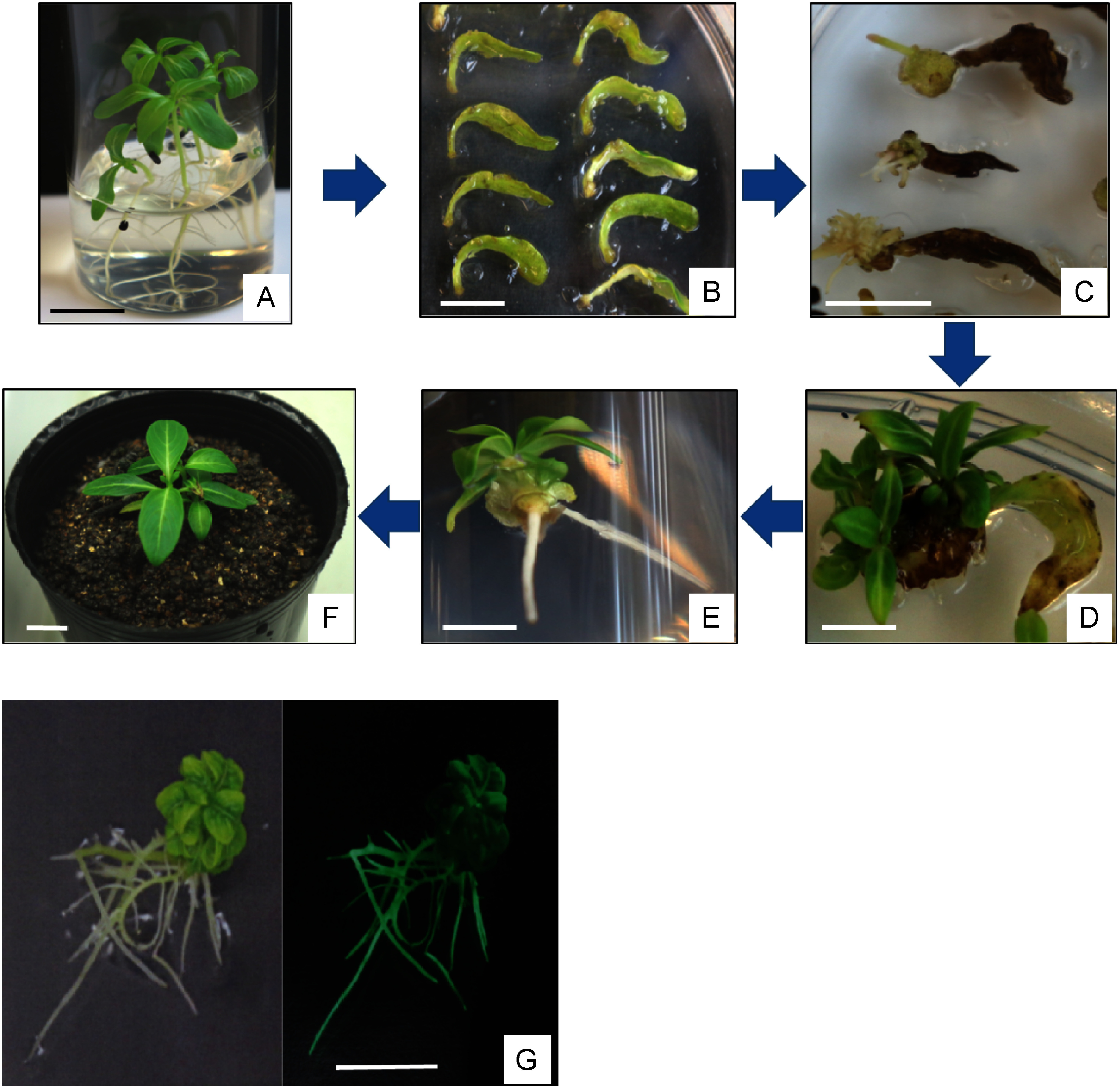
Figure 2. Process of transgenic plant production. (A) Aseptically sown seeds germinate and develop cotyledons. Cotyledons were carefully cut out and used as a material for infection. (B) Callus formation near the cut end of the cotyledon. (C) Root formation was confirmed about one month after *Agrobacterium* infection. (D) Shoot regenerated from the callus of the leaf piece that formed the root 1–2 months later. (E) Roots formed in medium containing antibiotics (100 mg l^−1^ kanamycin). (F) The regenerated plants acclimated to soil. (G) Observation of GFP fluorescence using regenerated plants infected with *Agrobacterium* carrying the GFP gene. Left: fluorescent lamp, right: blue light. Bar: 10 mm.

Cotyledons of 2-week-old seedlings aseptically seeded (Figure 2A) were excised, precultured in 1/2 B5 medium for 24 h, and infected by immersion in *Agrobacterium* suspension. After infection, cotyledons were cultured under photoperiod conditions at 25°C for 16 h. After 24 h, all cotyledons were transferred to 1/2 B5 medium containing 20 mg l^−1^ meropenem for sterilization of *Agrobacterium*. Furthermore, from day 2 post infection, cells were transferred to 1/2 B5 medium containing 50 mg l^−1^ kanamycin and 20 mg l^−1^ meropenem to initiate recombinant cell selection. Approximately one week after transplantation to the selective medium, calluses formed near the cut end of the cotyledon (Figure 2B), and we observed root formation one month later (Figure 2C). After another 1–2 months, shoots regenerated from calluses that differed from root-regenerated calluses (Figure 2D). Roots that regenerated first and shoots that regenerated later were derived from different calluses, whereby roots and shoots were not connected. Therefore, regenerated shoots were excised at the stem and transferred to 1/2 B5 medium containing 100 mg l^−1^ kanamycin to induce root regeneration (Figure 2E). Plants with formed roots that have acclimated to the soil and are used for genetic and component analyses (Figure 2F). By confirming the fluorescence of GFP using regenerated plants infected with *Agrobacterium* carrying the GFP gene, we were able to confirm the strong green fluorescence of GFP (Figure 2G).

### Producing transgenic plants using the JAED expression vector

**Figure 3:**
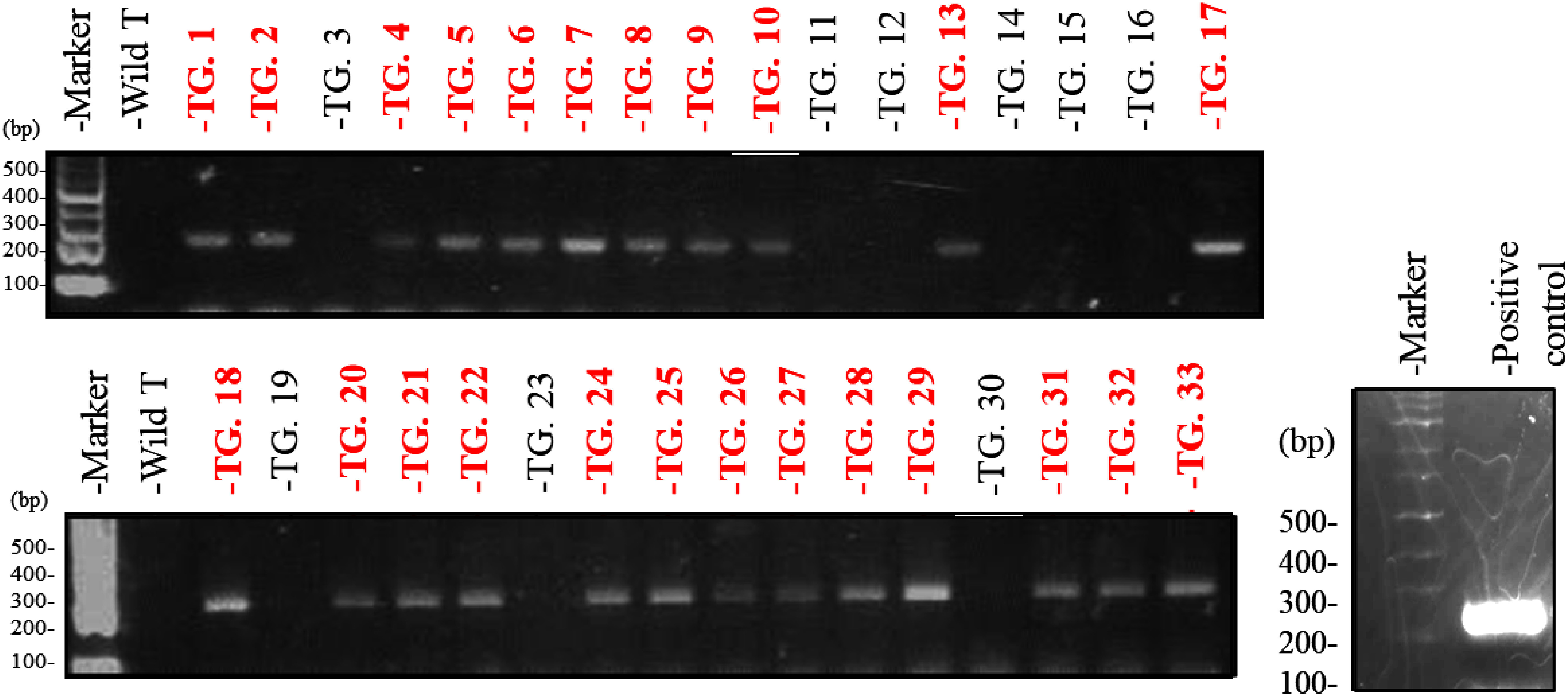
Figure 3. Genome PCR analysis using *NPTII* gene-specific primers. Amplification of bands was confirmed for lines with red letters. TG-1 to TG-33 indicated the numbers of regenerated plants. Wild T indicated wild-type *C. roseus*. A positive control was the result of performing a PCR reaction with the same primer set using the expression vector as a template.

Approximately 4,200 cotyledons were infected with *Agrobacterium* containing the JAED expression vector. We repeated selection on medium containing 50 mg l^−1^ kanamycin and 20 mg l^−1^ meropenem, resulting in the regeneration of 68 shoots. These shoots were transplanted to 1/2 B5 medium containing 100 mg l^−1^ kanamycin to induce rooting. As a result, 33 shoots formed roots. To confirm genes had been introduced into these regenerated plants, we performed genomic PCR analysis using primers specific to the *NPTII* gene, the kanamycin resistance gene. Therefore, we confirmed the presence of the *NPTII* gene in 24 of the 33 analyzed strains (Figure 3). The final transformation rate was 0.6% (Table 2). The morphology of the regenerated plants was very similar to the wild-type.

**Table 2:** Table 2. Transformation efficiency of *C. roseus* using our experimental methods.

	Number	Ratio (%)
Number of cotyledons infected with *Agrobacterium*	4,200	100
Number of regenerated shoots	68	1.6
Number of regenerated roots*	33	0.8
Number of strains confirmed for gene transfer	24	0.6

*Roots means obtained from regenerated shoots.

### Gene expression analysis of transgenic plants

**Figure 4:**
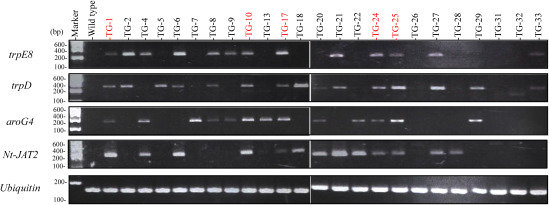
Figure 4. RT-PCR analysis. Total RNA was extracted from the strain in which the introduction of *NPTII* was confirmed (Figure 3), and cDNA was prepared from 1 µg of total RNA. PCR was performed using ubiquitin gene-specific primers to check the quality of the cDNA. Furthermore, PCR was performed using four gene-specific primers to confirm expression at the mRNA level. The primers used are described in the Materials and methods.

cDNA was prepared from the leaves of 24 JAED transgenic plants, and we used RT-PCR analysis to determine the transcriptional expression of each introduced gene. As a result, while some genes showed confirmed expression, others were not. We confirmed expression of only five transgenic plants—TG-1, TG-10, TG-17, TG-24 and TG-25—out of all the introduced genes. In TG-8, we confirmed expression of *trpE8*, *trpD*, and *aroG4* genes, but expression of *Nt-JAT2* was not confirmed. In addition, we selected TG-13 with *aroG4* and *Nt-JAT2* gene expression, TG-7 with *aroG4* expression, TG-6 with *trpE8*, *trpD*, and *Nt-JAT2* gene expression, and TG-2 and TG-33 with *trpE8* and *trpD* gene expression. These transgenic plants were used to measure tryptophan and alkaloid levels (Figure 4).

### Measuring free tryptophan content

**Figure 5:**
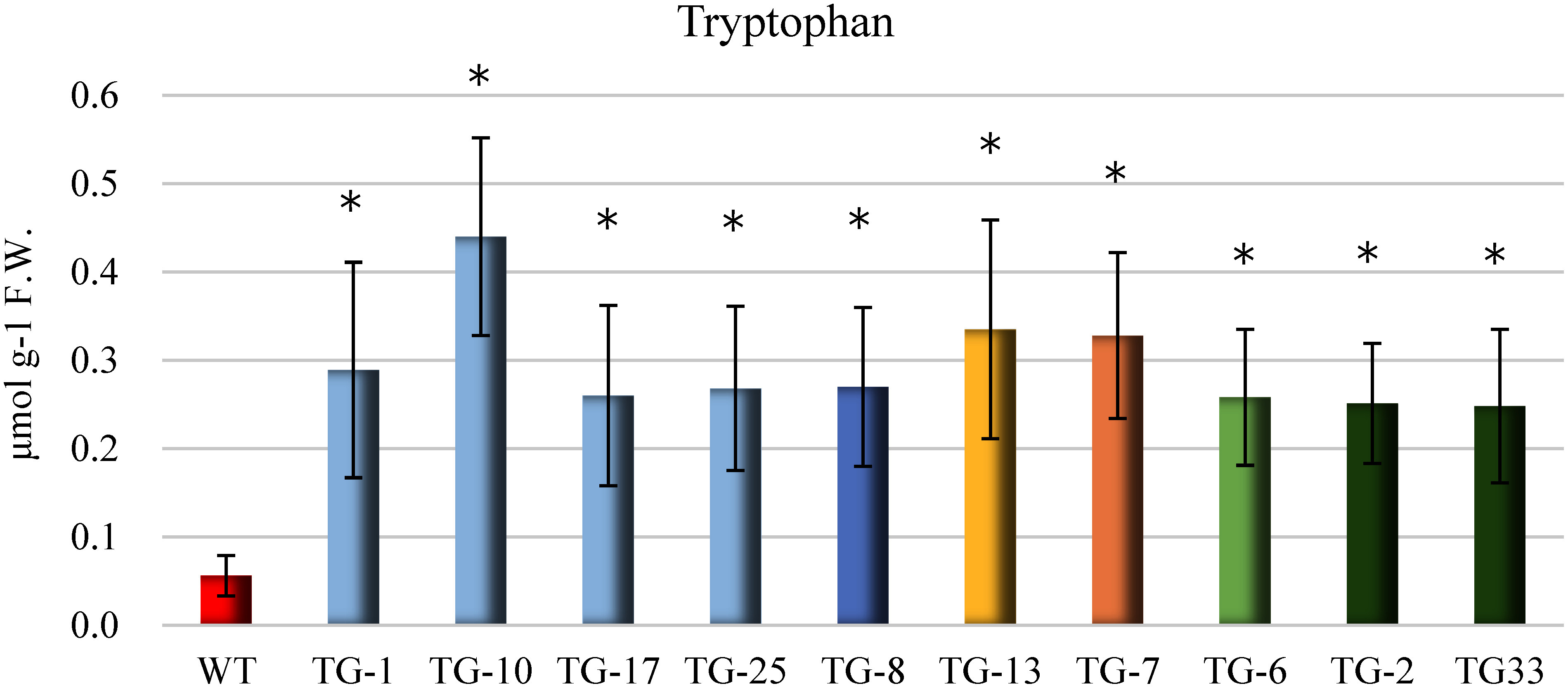
Figure 5. Free tryptophan content in the leaf tissue of JAED transformed plants (T_0_ generation). WT: wild-type *C. roseus*. We cut 100 mg leaf disks from each of the 4th, 5th, and 6th leaves from the growing point of the regenerated plant and sampled (*n*=3, *t*-test * *p*<0.05 ** *p*<0.01).

The free tryptophan content of wild-type *C. roseus* was 0.06 µmol g^−1^ F.W., but the transgenic plants showed a marked increase in tryptophan content. TG-10 showed the highest level at 0.44 µmol g^−1^ F.W., while TG-33 showed the lowest at 0.23 µmol g^−1^ F.W. (Figure 5). We confirmed an increase in tryptophan content not only in transgenic plants with confirmed expression of *trpE8* and *trpD* genes, but also in *aroG4* single-expression transgenic plants (TG-13 and TG-7).

### Measuring alkaloid content

**Figure 6:**
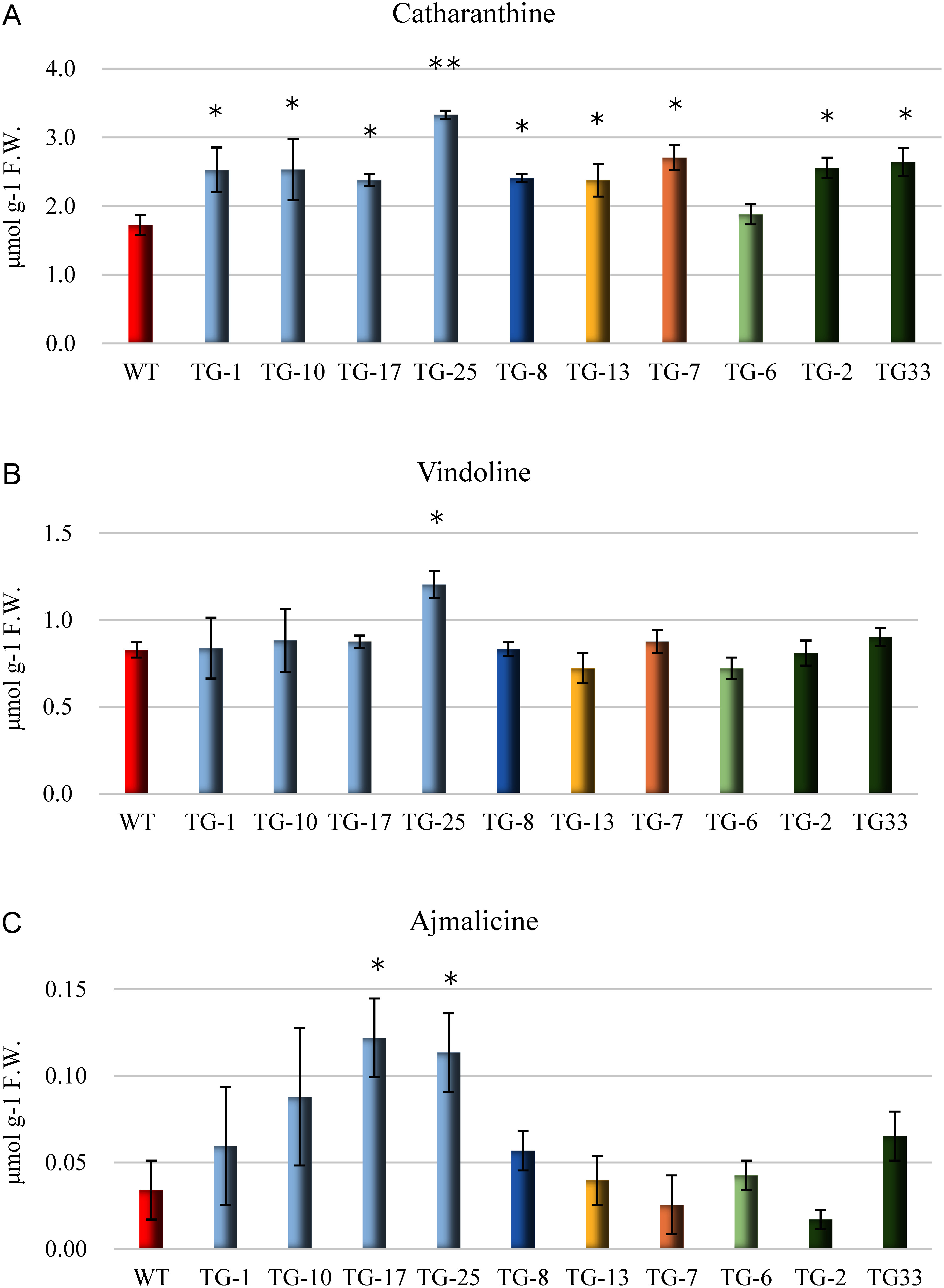
Figure 6. Alkaloid content in the leaf tissue of JAED transformed plants (T_0_ generation). WT: wild-type *C. roseus*. We cut 100 mg leaf disks from each of the 4th, 5th, and 6th leaves from the growing point of the regenerated plant as samples. (A) Catharanthine. (B) Vindoline. (C) Ajmalicine (*n*=3, *t*-test * *p*<0.05 ** *p*<0.01).

Catharanthine, vindoline, ajmalicine, and tabersonine contents were all measured using methanol extracts of leaves of wild-type and transformed plants. Tabersonine showed levels below the detection limit and could not be measured. Catharanthine content was 1.73 µmol g^−1^ F.W. for the wild-type, whereas transgenic plants showed a marked increase from 1.88 to 3.33 µmol g^−1^ F.W. The TG-25 line, which showed the most significant increase, had a statistically significant difference of 1% (Figure 6A). Vindoline content was 0.83 µmol g^−1^ F.W. for the wild-type. Among the transgenic plants, only TG-25 (1.20 µmol g^−1^ F.W.) showed a significant increase, and we observed no significant difference in other plants (Figure 6B). Ajmalicine levels in the wild strain were 0.03 µmol g^−1^ F.W., which was lower than catharanthine and vindoline. Among transgenic plants, we observed statistically significant increases in TG-17 and TG-25, but no significant differences in other lines (Figure 6C).

## Discussion

Several reports have examined the production of transformed plants of *Catharanthus roseus*. Wang et al. (2012) and Alam et al. (2017) produced efficient transgenic plants by sonicating cotyledons of *C. roseus* prior to infection with *Agrobacterium*. In this report, the optimal combination of plant hormones for shoot regeneration involved adding 5.0 mg l^−1^ 6-benzylaminopurine (6-BA) and 0.5 mg l^−1^ of naphthalene acetic acid. However, when *C. roseus* (cultivar, Pacifica XP) used in this study was cultured under the same plant hormone conditions, the cotyledons turned brown and slight calluses formed, but shoot regeneration was not achieved. For this reason, we investigated the types and combinations of plant hormones and medium composition used for culture conditions. As a result, cotyledon growth was better on the B5 medium containing no ammonium nitrogen and containing nitrate nitrogen as the main component compared with the MS medium (Murashige and Skoog 1962) containing a large amount of ammonium nitrogen. Furthermore, the medium in which the composition of B5 medium was diluted to half was the most suitable for cotyledon growth. In addition, as a result of examining plant hormones, we found green calluses when using the 1/2 B5 medium after adding 2 mg l^−1^ plant hormone *trans*-zeatin or when not added with plant hormones (called plant hormone free medium). Calluses in the plant hormone free medium were compact and hard, and we confirmed root and shoot regeneration in this medium. In addition, when commonly-used antibiotics, cefotaxime and carbenicillin, were used during sterilization of *Agrobacterium* when producing transgenic plants, cotyledons turned brown in about two weeks. *Agrobacterium* growth could not be inhibited with the medium containing these antibiotics after infection with *Agrobacterium*. Therefore, after examining how antibiotics can eradicate *Agrobacterium*, we found *Agrobacterium* growth was suppressed by adding 20 mg l^−1^ meropenem to the medium. Using meropenem for its inhibitory effect at low concentrations resulted in a long-term selective culture without browning of cotyledons.

In this study, we used an expression vector in which four gene cassettes were linked in tandem. Introduction of *NPTII* was confirmed in 24 transgenic plants, but the expression of all four genes could only be confirmed in five transgenic plants (TG-1, TG-10, TG-17, TG-24, and TG-25). Silencing occurred at the transcriptional level in many transgenic plants. Various changes were observed, such as complete silencing of certain genes and decreased expression of other genes. Because we used genes derived from microorganisms in this study, the possibility of co-suppression of exogenous genes and endogenous genes is considered low. Matzke and Matzke (1995) reported that methylation caused by interactions between foreign genes resulted in the regulation of gene expression at the transcriptional level. In addition, Smith et al. (1994) reported that mRNA accumulated in the nucleus is double-stranded RNA synthesized by an RNA polymerase and is then degraded by RNase. A decrease in the transcription of the introduced gene was also observed in this study. It is presumed that the reduction in mRNA expression occurred due to methylation of the introduced genes and degradation of RNA. Because it is possible that silencing is further increased by updating the generation of transgenic plants, we first performed amino acid and alkaloid analyses using the current transgenic plants (R_0_ generation) in which gene expression was confirmed. Unfortunately, the TG-24 plant had poor growth and died during cultivation.

Since there is no stable transformation system for *C. roseus*, functional analysis of alkaloid metabolism system genes using cultured cells and hairy roots of *C. roseus* has been reported. Whitmer et al. (1998) found the alkaloid content of cultured cells of *C. roseus* transformed with an increasing strictosidine synthase (STR) gene when feeding the alkaloid precursors tryptamine or loganin. From this result, they reported an inadequate supply of iridoid or indole precursors that limits metabolism catalyzed by strictosidine synthase. Verma et al. (2015) generated transformed cells introduced with *STR* and *TDC* genes and reported a doubling of total alkaloid content in these cultured cells compared with controls. Li et al. (2013) reported that metabolic analysis using hairy roots of *C. roseus* over-expressing the *ORCA2* gene showed ORCA2 regulates expression of *STR* and *SGD* genes and contributes to downstream regulation of the terpenoid-indole alkaloid pathway. In this study, we predicted that establishing a transformation system that stably introduces genes will be useful for analyzing the functions of alkaloid metabolism genes in plants and accumulating useful components.

Hughes et al. (2004) used a dexamethasone (DEX)-inducible vector to transform the AS alpha subunit isolated from *Arabidopsis thaliana* into the hairy roots of *C. roseus*. Induced gene expression with DEX resulted in a maximum 300-fold increase in tryptophan content compared with controls and a 1.8-fold increase in lochnericine content, affecting alkaloid content. In our study, an increase in catharanthine was confirmed with an increase in tryptophan. Catharanthine is synthesized in leaves and stems, but barely synthesized in cultured cell or hairy roots. Chang et al. (2014) reported that the amount of catharanthine contained in the roots of *C. roseus* was about 1/25 of that contained in the leaves. Therefore, that study may not have found an effect on catharanthine. Since the transgenic plants were obtained, it is clear that catharanthine increased in the leaves of the plants. This study is the first report showing that increasing tryptophan content significantly increased catharanthine in *C. roseus* leaves. Peebles et al. (2005) combined a feedback-deregulated AS alpha subunit gene isolated from *Arabidopsis thaliana* into an inducible expression vector and introduced it into *C. roseus* hairy roots. Within 72 h of induction, the results showed increased tryptophan, tryptamine, and ajmalicine concentrations and reduced tabersonine concentrations in hairy roots. Further, some transgenic plants with increased tryptophan content helped increase ajmalicine content. Roepke et al. (2010) reported that some catharanthine produced in *C. roseus* epidermal cells condenses with vindoline, and it is used to synthesize vinblastine and vincristine. Moreover, when *C. roseus* hairy roots over-expressing the AS gene were fed with the terpenoid precursor loganin, the downstream alkaloids catharanthine (26%), ajmalicine (84%), lochnericine (119%), and tabersonine (225%) significantly increased compared with unsupplemented hairy roots (Peebles et al. 2006), but neither case affected vindoline content.

In this study, we confirmed a significant increase in the tryptophan content of transgenic plants. In particular the results of RT-PCR analysis suggested that co-expression of *trpE8* and *trpD* genes or single expression of the *aroG4* gene significantly increased the tryptophan content. These results suggest that the feedback resistance function of tryptophan in microorganisms operates even when introduced into plants.

Furthermore, in transgenic plants with high tryptophan content, we also detected increased catharanthine levels, but no significant increase was observed in vindoline levels. Catharanthine and vindoline are synthesized from strictosidine produced by the polymerization of tryptamine and secologanin. Furthermore, strictosidine is metabolized to strictosidine glucose, 4,21-dehydrogeissoschizine, and stemmadenine, branching into the synthetic pathway of catharanthine and synthetic pathway of vindoline. Qu et al. (2015) reported that seven enzymes are involved in the biosynthesis of vindoline from tabersonine, and T16H, NMT, and D4H activities might be flux-limiting components of the pathway. Because the vindoline content remained mostly unchanged in this study, it thought that the vindoline metabolic pathway is tightly regulation. Furthermore, an increase in ajmalicine content was confirmed in some transgenic plants. Ajmalicine is also produced from strictosidine; however, there are few reports regarding the partitioning of 4,21-dehydrogeissoschizine into the ajmalicine and stemmadenine metabolic pathways. Elevated tryptophan metabolism might have increased the metabolic flux, and some of the tryptophan may have been utilized in ajmalicine metabolism. However, the control of this regulation requires further investigation for elucidation. Both vinblastine and vincristine content were below the detection limits under these experimental conditions. In the future, we plan to use LC-MS analysis for this purpose. The alkaloid transporter Nt-JAT2 used in this study. However, there was no difference in alkaloid content between transgenic plants with confirmed *Nt-JAT2* gene expression and those in which *Nt-JAT2* gene expression was not confirmed (e.g., TG-1 and TG-8, TG-13 and TG-7, and TG-2 and TG-6). The Nt-JAT2 failed to transport alkaloids in *C. roseus*. Reasons for non-functioning of Nt-JAT2 transporter include different specification of transporter for their substrates and different localization of substrates and transporter. *CrTPT2* is a known alkaloid transporter in *C. roseus* that transports catharanthine to the epidermal surface and secretes it (Yu and De Luca 2013), which may contribute to the efficient accumulation of catharanthine. Further research in this area is warranted.

## Abbreviations

aroG: 3-deoxy-D-arabino-heptulosonate-7-phosphate synthase
AS: anthranilate synthase
D4H: desacetoxyvindoline-4-hydroxylase
*E. coli*: *Escherichia coli*
NMT: N-methyltransferase
NPF: nitrate/peptide family
TIAs: terpenoid-indole alkaloids
T16H: tabersonine 16-hydroxylase
